# Nitrous Oxide

**Published:** 1872-05

**Authors:** 


### Nitrous Oxide.

Mrs. Annie O'Shaughnessy, of New York City, was killed on the 20th ult., by inhaling Nitrous Oxide Gas, taken to make the extraction of some teeth painless. Mrs. O'Shaughnessy was regarded as ordinarily healthy—age 35 years.

Death from the use of anæsthetics in dental practice, is becoming a common occurrence, and why people will persist in their use at the risk of life, is to us the greatest of mysteries.

The indiscriminate and reckless manner in which nitrous oxide or laughing gas is used, makes it the more surprising that the deaths are not more frequent, for the fact that its administration is attended with great risk to life, is not only substantiated by the death of Mrs. O'Shaughnessy, and numerous other cases reported, but it is the opinion of the highest medical authority of the land, that the use of chloroform, ether nitrous oxide, and all general anæsthetics is attended with a certain degree of mortality.

There are many conditions in which nitrous oxide is contra-indicated, among which may be mentioned a derangement of the heart, a predisposition to congestion, or inflammation of the brain, and diseases of the lungs and kidneys, etc.

If it were possible to determine when these conditions exist in every case, than by careful diagnosis and necessary care, much of the danger might be averted, but unfortunately it is a physiological fact that there are certain conditions in which the administration of anæsthetics is inadmissible, and that are positively impossible to determine. Chloroform depresses the nervous system, and consequently vital action is reduced below its normal standard. Nitrous oxide, on the other hand, stimulates and elevates the nervous system, and carries vital action above its normal standard. The result of either may be the same, and the danger, we think, may be equally balanced. If there be a difference, carelessness, recklessness, and ignorance in the use of nitrous oxide will outweigh all its "life supporting claim," and the speedy recovery from its affects, or any other advantage it may claim over chloroform. Hundreds who use nitrous oxide with impunity, would not under any consideration, dare to tamper with chloroform.

We desire to be understood as objecting to general anæsthetics only in minor operations, such as occasion only momentary pain, and not objecting where the cases are capital in character. Let us examine ourselves carefully and thoroughly, and if we are determined to use an anæsthetic for the purpose of alleviating pain incident to a simple operation in dental surgery, let us be sure before incurring such risk, that we are in proper physical condition, and that the operators are sufficiently competent and skillful.

